# Hepatitis E in 24 Chinese Cities, 2008–2018: A New Analysis Method for the Disease's Occupational Characteristics

**DOI:** 10.3389/fpubh.2021.720953

**Published:** 2021-09-28

**Authors:** Shanshan Yu, Jia Rui, Xiaoqing Cheng, Zeyu Zhao, Chan Liu, Shengnan Lin, Yuanzhao Zhu, Yao Wang, Jingwen Xu, Meng Yang, Xingchun Liu, Mingzhai Wang, Zhao Lei, Benhua Zhao, Qinglong Zhao, Xuefeng Zhang, Tianmu Chen

**Affiliations:** ^1^State Key Laboratory of Molecular Vaccinology and Molecular Diagnostics, School of Public Health, Xiamen University, Xiamen, China; ^2^Jiangsu Provincial Center for Disease Control and Prevention, Nanjing, China; ^3^Xiamen City Center for Disease Control, Xiamen, China; ^4^Jilin Provincial Center for Disease Control and Prevention, Changchun, China

**Keywords:** HEV, occupational classification, new method, cluster analysis, diversity, similarity

## Abstract

**Background:** The disease burden of hepatitis E remains high. We used a new method (richness, diversity, evenness, and similarity analyses) to classify cities according to the occupational classification of hepatitis E patients across regions in China and compared the results of cluster analysis.

**Methods:** Data on reported hepatitis E cases from 2008 to 2018 were collected from 24 cities (9 in Jilin Province, 13 in Jiangsu Province, Xiamen City, and Chuxiong Yi Autonomous Prefecture). Traditional statistical methods were used to describe the epidemiological characteristics of hepatitis E patients, while the new method and cluster analysis were used to classify the cities by analyzing the occupational composition across regions.

**Results:** The prevalence of hepatitis E in eastern China (Jiangsu Province) was similar to that in the south (Xiamen City) and southwest of China (Chuxiong Yi Autonomous Prefecture), but higher than that in the north (Jilin Province). The age of hepatitis E patients was concentrated between 41 and 60 years, and the sex ratio ranged from 1:1.6 to 1:3.4. Farming was the most highly prevalent occupation; other sub-prevalent occupations included retirement, housework and unemployment. The incidence of occupations among migrant workers, medical staff, teachers, and students was moderate. There were several occupational types with few or no records, such as catering industry, caregivers and babysitters, diaspora children, childcare, herders, and fishing (boat) people. The occupational similarity of hepatitis E was high among economically developed cities, such as Nanjing, Wuxi, Baicheng, and Xiamen, while the similarity was small among cities with large economic disparities, such as Nanjing and Chuxiong Yi Autonomous Prefecture. A comparison of the classification results revealed more similarities and some differences when using these two methods.

**Conclusion:** In China, the factors with the greatest influence on the prevalence of hepatitis E are living in the south, farming as an occupation, being middle-aged or elderly, and being male. The 24 cities we studied were highly diverse and moderately similar in terms of the occupational distribution of patients with hepatitis E. We confirmed the validity of the new method on in classifying cities according to their occupational composition by comparing it with the clustering method.

## Introduction

Hepatitis E virus (HEV) infection is a great economic burden on Chinese people. Data released by the Chinese Centre For Disease Control and Prevention show that from 2010 to 2020, the proportion of HEV cases among all infectious diseases was one in one thousand (1), especially in rural areas (2). The results of a health economics study in Jiangsu Province showed that the total economic burden of HEV cases accounted for 60.77% of the per capita disposable income (3). Genotypes 1 and 2 are responsible for the majority of acute viral hepatitis infections in endemic areas in South Asia (4), which are limited to humans and non-human primates and have been found in areas with frequent water contamination via fecal-oral transmission, mostly in developing countries with limited access to sanitation. Genotypes 3 and 4 are related to zoonotic diseases, being low-endemic in developed countries, and transmitted by eating infected animal meat or having close contact with animals (5). The World Health Organization (WHO) has set the global target to reduce new viral hepatitis infections by 90% and reduce deaths due to viral hepatitis by 65% by 2030 (6). Therefore, research on HEV has public health significance.

The relationship between occupation and HEV susceptibility remains unclear. Risky occupational populations are present. Studies in Moldova (7) and Cuba (8) and Accra, Ghana (9, 10) have shown that the detection rate of HEV antibodies in people with occupational exposures, such as pig workers, is higher than that in the general non-occupational population. In China, one study found certain occupations to be more at risk for being frequently exposed to pathogens, such as people working in the catering industry, livestock breeders, soldiers, field workers, college students, and migrant workers or business travellers in epidemic areas (11). Another epidemiological survey showed that additional occupational populations at risk are farmers, retirees, domestic workers, and unemployed people (12). Several studies on pig slaughtering and sales workers in Zhejiang Province and Shanghai Municipality of China have confirmed this (13, 14). However, another study in Zhejiang Province showed that the infection rate of HEV among pig slaughterers and pet breeders is not different from that of the general population (15). Existing surveys have the limitations of small sample sizes and restricted survey areas. Therefore, our study aimed to investigate the occupational characteristics of HEV cases in 24 cities across 18 occupations based on the Infectious Disease Report Card of the People's Republic of China. Our objective was to better understand the differences in the occupational classification of HEV cases in China.

Cluster analysis has been proven to be an effective classification method when conducting age stratification of patients with diabetes (16), studying differences in the degree of environmental pollution (17), and recognizing temperature zones in China (18).

The new method (19) was also used for the classification. Previously, we used this new method to analyse the species composition and similarity of malaria vectors (20), and found that the sub-regions of Changsha City shared moderate diversity and high similarity for occupational distribution of hand, foot, and mouth disease (21). The new method supplements the traditional descriptive analysis method and contains six indicators, including richness, diversity, evenness, and similarity analyses. Although the feasibility of this new method has been verified in the above studies, the effectiveness of this classification method has not been verified. In this paper, we compare the classification results of the new method with those of the clustering analysis by analyzing the HEV occupational incidence.

In summary, we aimed to investigate the characteristics of occupational distributions in interregional HEV patients. In addition, we hope to confirm the validity of the new method in classification analysis by comparing it with the clustering analysis.

## Methods

### Study Design

**Figure 1:**
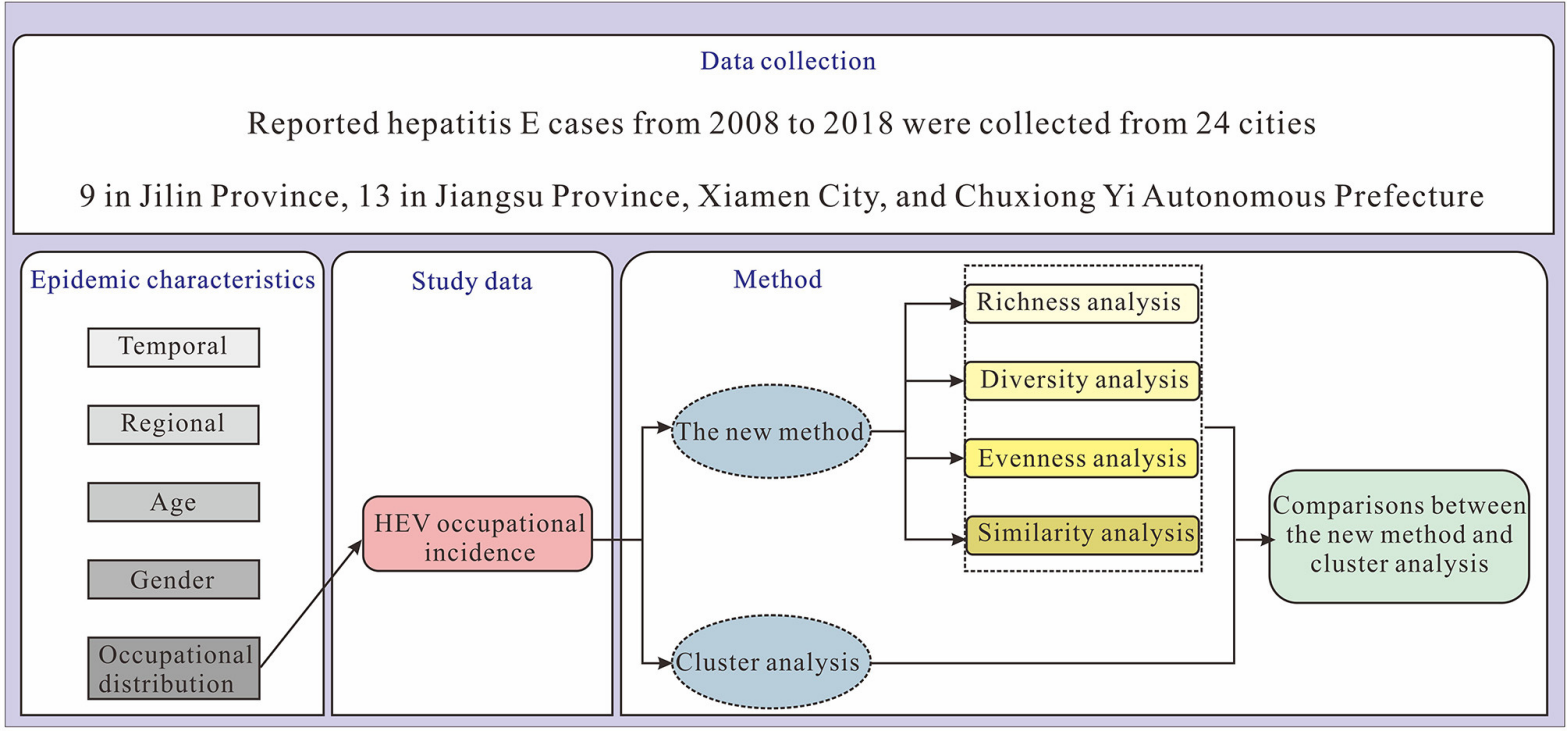
Research design flow diagram.

This study is divided into four sections. The first section provides a brief overview of HEV epidemiological characteristics in terms of temporal, regional, age, gender, and occupational distributions of reported HEV cases. In the second section, the new method is used for richness, diversity, evenness, and similarity analyses. The results of the cluster analysis are presented in the third section. The final section analyzes the results of classifying cities using the new method and cluster analysis. A research flow diagram is shown in Figure 1.

### Study Areas

**Figure 2:**
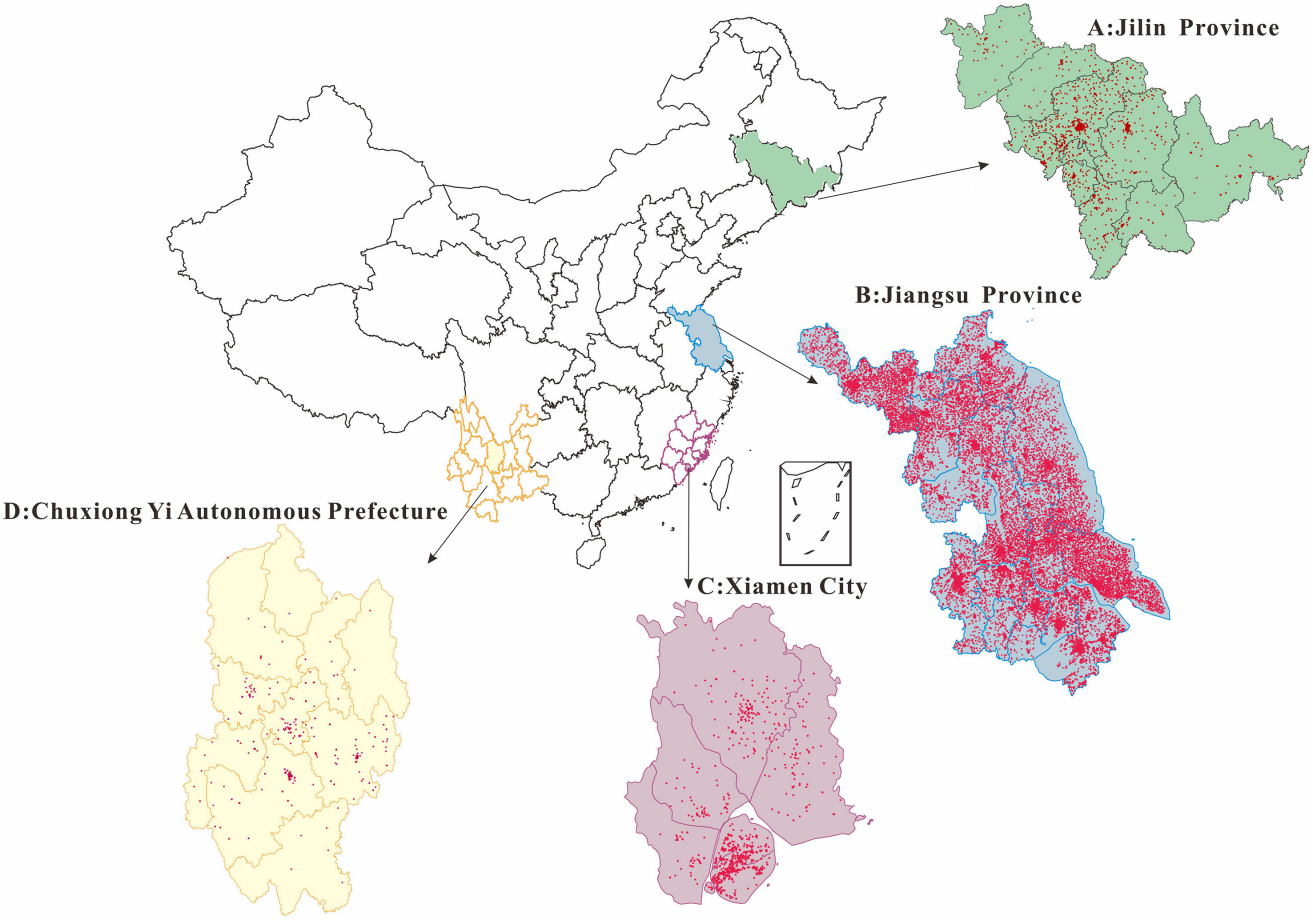
Punctuation map of incidence in our study areas (2008–2018). **(A)** Jilin Province (in the north of China). **(B)** Jiangsu Province (in the east of China). **(C)** Xiamen City (in the south of China). **(D)** Chuxiong Yi Autonomous Prefecture (in the southwest of China). The map depicted in this figure was taken from Wikimedia Commons (http://commons.wikimedia.org/wiki/Main_Page).

We selected 24 cities in northern, eastern, southern, and southwestern China as the study area (Figure 2). To compare the difference among occupations of patients with HEV between the north and the south, we used data from Jilin Province in the north of mainland and Jiangsu Province in the south of mainland. Furthermore, we compared the differences within provinces; therefore, we separately compared and analyzed the occupational incidence of HEV infection in nine cities in Jilin Province and 13 cities in Jiangsu Province. In addition, to compare the differences among different cities, we added the data from Xiamen City of Fujian Province and Chuxiong Yi Autonomous Prefecture of Yunnan Province to those of the 22 cities under Jilin and Jiangsu Provinces for comparative analysis.

Jilin Province (40°52–46°18′*N*, 121°38–131°19′*E*, northern) has jurisdiction over nine prefecture-level administrative regions, including eight prefecture-level cities and one autonomous prefecture (Changchun City, Jilin City, Siping City, Liaoyuan City, Tonghua City, Baishan City, Songyuan City, Baicheng City, and Yanbian Korean Autonomous Prefecture).

Jiangsu Province (30°45–35°20′*N*, 116°18–121°57′*E*, eastern) governs 13 prefecture-level administrative regions (The southern part: Nanjing City, Suzhou City, Wuxi City, Changzhou City, Zhenjiang City; The central part: Yangzhou City, Taizhou City, Nantong City; The northern part: Xuzhou City, Huaian City, Suqian City, Lianyungang City, Yancheng City).

Xiamen City (24°26′*N*, 118°04′*E*, southern), with a total area of 170,061 square kilometers, is located at the southeast end of Fujian Province.

Chuxiong Yi Autonomous Prefecture (24°13–26°30′*N*, 100°43–102°30′*E*, southwestern) is located in the middle of Yunnan Province, with a total area of 29,000 square kilometers.

### Data Collection

A dataset of symptomatic cases of HEV reported in 24 cities from 2008 to 2018 was created, including date of onset and type of occupation, age, sex, current address, disease classification, and excluding disease severity. In China, hepatitis B and C cases are classified as acute or chronic. However, hepatitis E patients were all acute, and the disease classification column of our dataset was unclassified, which actually refers to acute hepatitis E. Disease data were obtained from the Centre for Disease Control and Prevention of Jilin Province, Jiangsu Province, Xiamen City, and Chuxiong Yi Autonomous Prefecture separately. Demographic data for the 24 cities were obtained from the National Statistics Bureau.

Occupational classification was based on the Infectious Disease Report Card of the People's Republic of China stipulated in the Law of the People's Republic of China on prevention and control of infectious diseases (22), which came into force on December 1, 2004. The 18 occupations were classified as childcare (for kindergarten children), diaspora children (for children raised at home, who have not yet been to school), students (including students in primary, secondary, and high school or in college), teachers, caregivers and babysitters, catering industry, business services, medical staff, workers, migrant workers (farmers working outside of their town of origin), farmers, herders, fishing (boat) people, cadre staff, retiree, housework and unemployment, others, and unknown.

### Diagnostic Criteria

According to the “Code of Practice for the Treatment of Viral Hepatitis E” formulated by the Infectious Disease Physicians Branch of the Chinese Medical Association in July 2009 (23), a comprehensive diagnosis was made based on the epidemiological history, symptoms, signs, and laboratory examination results. Diagnostic criteria for acute HEV infection include positive anti-HEV IgM, 4-fold or greater increase in anti-HEV IgG positive conversion or content, and positive serum and/or fecal HEV RNA. In general, the positive results of any of these three indicators can be used as the basis for the clinical diagnosis of acute HEV infection, and the diagnosis can be confirmed if multiple indicators have positive results. Anti-HEV IgM detection reagents developed at home and abroad relying on conformational antigens have been developed (24), and anti-HEV IgM has become the most important diagnostic indicator of acute HEV infection in clinical practice; IgM antibody positivity and IgG antibody positivity can generally be diagnosed.

### Statistical Methods

First, the traditional descriptive epidemiological method was adopted to analyze the temporal and regional, age and gender, and occupational distributions of the reported cases. Second, the new method (19) was used to describe the similarity and diversity of HEV, including six indices: richness index (*N*), Simpson diversity index (*D*), Shannon diversity index (*H*), Berger–Parker dominance index (*d*), Shannon evenness index (*E*), and Morisita–Horn similarity index (*C*). *N* represents the number of occupational classifications. The *p*_*i*_ refers to the proportion of the *ith* classification, and the maximum of *p*_*i*_ is the index *d*, which measures occupational dominance. Occupational diversity and evenness were evaluated using three indices: *D, H*, and *E*. If *D* is closer to 1 or *H* is larger, the diversity will be greater. The closer *E* is to 0.5, the better the equitability. The larger *d* is, the stronger the dominant occupation. Similarities among the different study areas was measured using index *C*. The closer *C* is to 1, the greater the similarity. The indices *D* and *H* were calculated from the proportion of each occupation; *E* was calculated by dividing *H* by the richness index, and *C* was calculated by the number of individuals in each occupation and the total number of populations by region. These indices are represented by the following equations:


$$\begin{array}{l} D=1-\sum_{n=1}^{N}p_{i}^{2}\\ H=1\sum_{n=1}^{N}p_{i}\ln p_{i}\\ \;E=\frac{H}{\ln N}\\ C=\frac{2\sum n_{1i}n_{2i}}{\left(\lambda_{1}+\lambda_{2}\right)M_{1}M_{2}},\lambda_{i}=\frac{\sum n_{ji}^{2}}{M_{j}^{2}} \end{array}$$

Third, we used the cluster analysis method (16), which is a multivariate statistical analysis method. The between-group linkage method was used to calculate the distance between classes. By comparing the properties of various samples, those with similar properties are classified into one category, and those with different properties are divided into different categories (25). The clustering method regards *N* samples as *N* classes at the beginning, and then merges them step by step until *N* samples are merged into one class. In this study, each of the 24 study cities was regarded as a sample, and clustering was carried out according to the incidence of 18 occupational reports (for example, farmers, students, herders) of patients with HEV from 2008 to 2018.

Microsoft Excel 2019 software (Microsoft Corp, USA) was used for data entry, sorting, drawing, and calculation of the six indices. The data were analyzed using IBM SPSS Statistics for Windows, version 26.0 (IBM Corp., Armonk, N.Y., USA) for *Q*-type clustering analysis. The statistically significant level was set at *P* < 0.05. DataMap 6.2 software (Microsoft Corp, USA), was used to create punctuation maps.

## Results

### Distributions of Traditional Descriptive Epidemiological Method

#### Temporal and Regional Distributions of Reported HEV Cases

**Figure 3:**
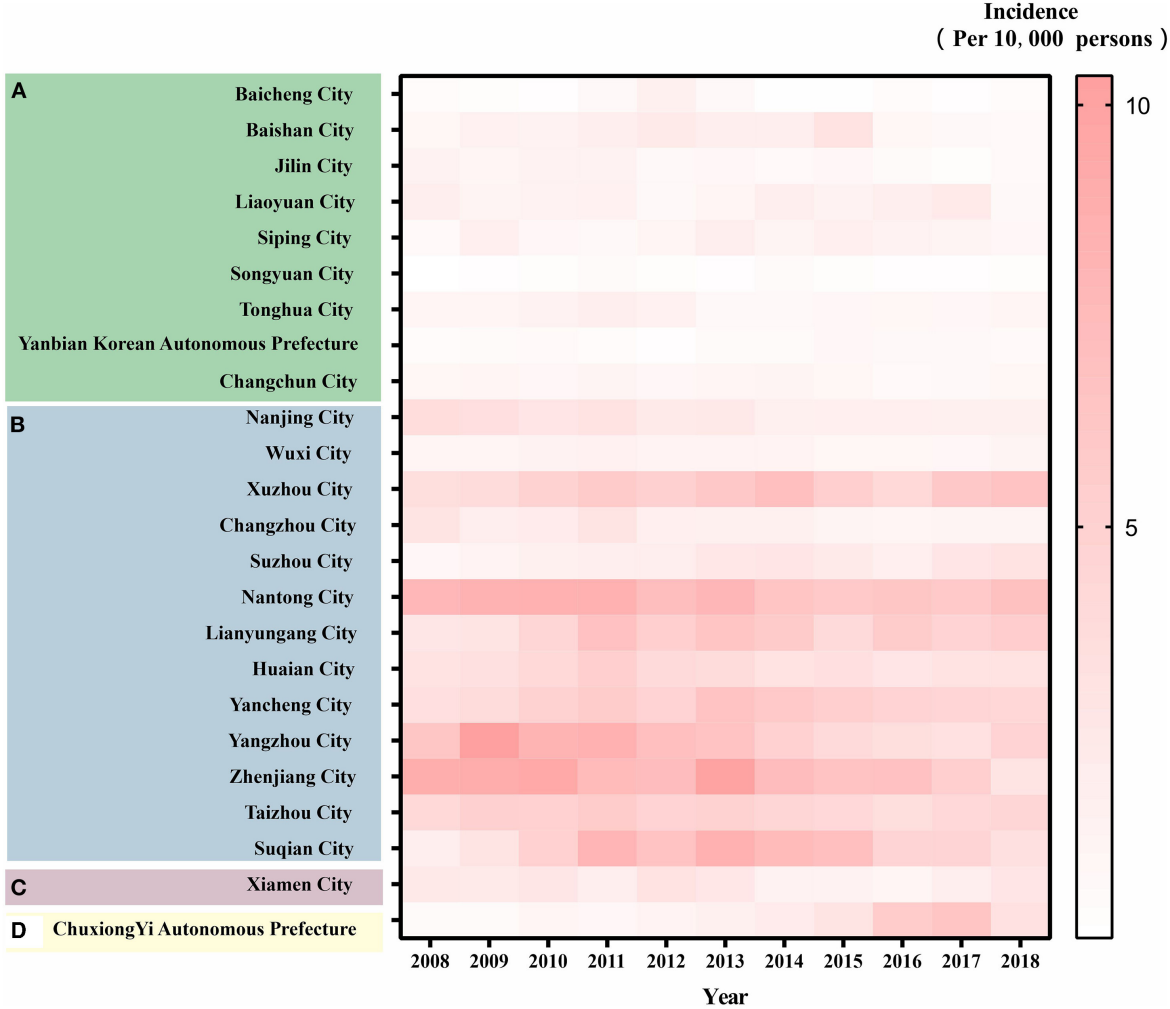
Heat map of reported incidence of HEV cases in 24 cities (2008-2018). **(A)** Jilin Province (in the north of China). **(B)** Jiangsu Province (in the east of China). **(C)** Xiamen City (in the south of China). **(D)** Chuxiong Yi Autonomous Prefecture (in the southwest of China). The following is the same.

Figure 3 depicts the incidence of HEV infection in 24 cities from 2008 to 2018. The incidence of HEV infection in northern China (Jilin Province) was similar to that in western China (Chuxiong Yi Autonomous Prefecture) and southern China (Xiamen City) but was lower than that in southern China (Jiangsu Province).

We found that the dynamics of prevalence varied within the provinces. In Jilin Province, the incidence of HEV in the four cities increased, while that in the other five cities showed an annual decreasing trend. In Jiangsu Province, with the exception of the incidence of HEV in the two cities that were on the rise, the other 11 cities showed a downward trend. The incidence in Xiamen City, a coastal city, has been increasing annually, while the incidence in Chuxiong Yi Autonomous Prefecture, an inland city, is decreasing.

#### Age and Gender Distributions of Reported Incidence of Patients With HEV

**Figure 4:**
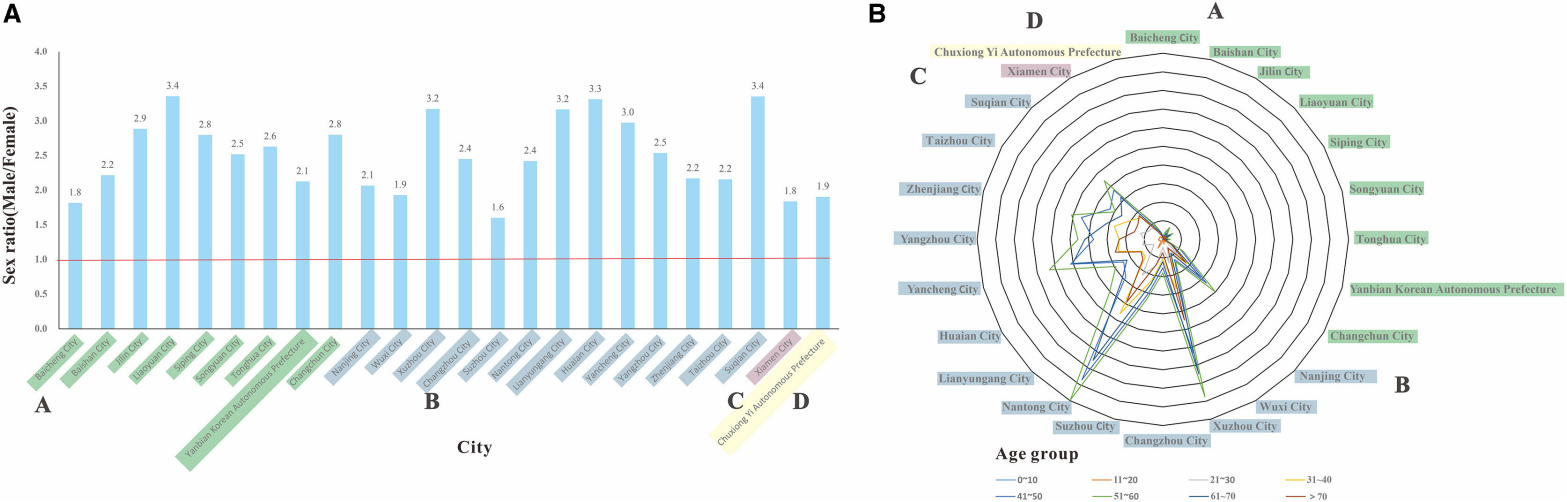
Sex and age distribution of reported HEV cases in 24 cities (2008-2018). **(A)** sex ratio distribution of the study regions. **(B)** distribution of HEV cases across the study regions by age groups.

According to the radar map of age distribution in the 24 cities (Figure 4), the majority of patients with HEV were in the 41–50 and 51–60 age ranges. The proportions for the 60–70 years and the 31–40 years age groups were medium. The proportion of those over 70 years of age and under the age of 20 years was lower.

In terms of sex, there were significantly more males than females (Figure 4), and the sex ratio ranged from 1.6 to 3.4, with the highest sex ratio of 3.4, in Suqian City, Jiangsu Province, and the lowest sex ratio of 1.6, in Suzhou City, Jiangsu Province.

#### Occupational Distribution of Reported HEV Cases

Table 1 shows the percentage of cumulative HEV cases in 18 occupations. In summary, farmers accounted for the largest proportion of occupation types among all cities, followed by housework and unemployment, retirees, and workers. The top two occupational types among HEV cases in Jilin Province were farmers and retirees. Among the 13 cities in Jiangsu Province, farmers were the highest occupational type, followed by houseworkers and the unemployed, retirees and workers. We found that the main occupation types in Xiamen City were the other types (not the above-mentioned 17 types) and farmers. Farmers and retirees accounted for the highest proportions in the Chuxiong Yi Autonomous Prefecture of Yunnan Province. For 24 cities, we observed few or no records of caregivers and babysitters among the patients, and few cases were seen among diaspora children, childcares, herders, and fishing (boat) groups.

**Table 1:** Occupational distribution of reported HEV cases in 24 cities (2008–2018).

**City**	**Baicheng City**	**Baishan City**	**Jilin City**	**Liaoyuan City**	**Siping City**	**Songyuan City**	**Tonghua City**	**Yanbian Korean Autonomous Prefecture**
**Occupation**								
Business services	4	3	12	1	3	2	7	3
Cadre staff	7	13	27	14	11	5	13	14
Caregiver and babysitter	0	0	0	0	0	1	1	0
Catering industry	0	1	2	3	1	1	1	0
Childcare	0	0	0	0	0	0	0	0
Diaspora children	0	0	1	1	0	0	0	0
Farmer	39	28	158	48	233	60	115	23
Fishing (boat) people	0	0	0	0	0	0	0	0
Herder	0	0	1	1	1	0	0	0
Housework and unemployment	26	61	76	66	90	10	53	53
Medical staffs	1	2	2	2	3	0	0	1
Migrant workers	0	1	5	2	2	1	2	1
Others	8	17	3	11	10	11	18	22
Retired	21	39	82	26	33	6	21	16
Student	1	1	4	1	1	1	4	2
Teacher	0	6	6	2	0	0	3	2
Unknown	5	28	5	2	60	9	23	3
Worker	9	26	52	29	15	2	29	13

### The Results of the New Method on Occupational Types in the 24 Cities

#### The Richness Analysis by the New Method Using Index N

The *N*-value was highest in Xuzhou City (*N* = 17) and Lianyungang City (*N* = 17) of Jiangsu Province and was the lowest in Suzhou City (*N* = 2) and Nantong City (*N* = 2) of Jiangsu Province. There were more than 10 occupational types among patients with HEV in other cities, such as Jilin City (*N* = 16) in Jilin Province, Xiamen City (*N* = 14), and Chuxiong Yi Autonomous Prefecture (*N* = 14; Table 2).

**Table 2:** Analysis of richness, diversity and uniformity of 24 cities (2008–2018).

**Year**	**N**	**Diversity index**		**d**	**E**
		**D**	**H**		
Baicheng City	10	1.000	0.203	0.255	0.088
Baishan City	12	0.993	0.750	0.304	0.302
Jilin City	16	0.999	0.312	0.665	0.113
Liaoyuan City	15	0.805	1.887	0.462	0.697
Siping City	13	0.992	0.522	0.999	0.203
Songyuan City	12	1.000	0.085	1.000	0.034
Tonghua City	13	0.998	0.432	0.588	0.169
Yanbian Korean Autonomous Prefecture	12	0.999	0.321	0.767	0.129
Changchun City	12	0.993	0.679	0.734	0.273
Nanjing City	15	0.921	1.726	0.255	0.637
Wuxi City	14	0.887	1.758	0.304	0.666
Xuzhou City	17	0.653	1.063	0.665	0.375
Changzhou City	16	0.732	1.728	0.462	0.623
Suzhou City	2	0.235	0.124	0.999	0.179
Nantong City	2	0.001	0.002	1.000	0.004
Lianyungang City	17	0.702	1.408	0.588	0.497
Huaian City	16	0.541	1.061	0.767	0.383
Yancheng City	16	0.595	1.104	0.734	0.398
Yangzhou City	16	0.695	1.274	0.646	0.459
Zhenjiang City	16	0.866	1.408	0.448	0.508
Taizhou City	16	0.669	1.343	0.633	0.484
Suqian City	14	0.472	0.974	0.780	0.369
Xiamen City	14	0.991	0.929	0.780	0.352
Chuxiong Yi Autonomous Prefecture	14	0.993	0.379	0.780	0.144

#### The Diversity Analysis by the New Method Using Index D and H

In Jilin Province, the Simpson diversity index (*D* = 0.805) and Shannon diversity index (*H* = 1.887) of Liaoyuan City were the highest. In Jiangsu Province, the two indices were the highest in Nanjing City (*D* = 0.921, *H* = 1.726) and the lowest in Nantong City (*D* = 0.001, *H* = 0.002). In Xiamen City, Fujian Province, the Simpson diversity index (*D* = 0.991) was close to 1, while the Shannon diversity index (*H* = 0.929) was high. The Simpson diversity index (*D* = 0.993) of Chuxiong Yi Autonomous Prefecture in Yunnan Province was close to 1, while the Shannon diversity index (*H* = 0.379) was low (Table 2).

#### The Evenness Analysis by the New Method Using Index E and d

The evenness index of most cities in Jiangsu Province was close to 0.5, for example, Lianyungang City (*E* = 0.497), Zhenjiang City (*E* = 0.508), and Taizhou city (*E* = 0.484). However, the *E*-values of Nantong City (*E* = 0.004) and Suzhou City (*E* = 0.179) were the opposite. All nine cities in Jilin Province (*E* = 0.088–0.697) were away from 0.5. Similarly, the same was true for Xiamen City (*E* = 0.352) and Chuxiong Yi Autonomous Prefecture (*E* = 0.144; Table 2).

The farmer group was the occupational type with the largest Berger-Parker dominance index *d* in most cities, such as Siping City, Suzhou City, and Nantong City (*d* = 0.999–1.000). However, the *d*-values of Baicheng City (*d* = 0.255) and Nanjing City (*d* = 0.255) were much lower.

The similarity analysis by the new method using index *C* Table 3 shows the similarity coefficient matrix of HEV infection among the 24 cities from 2008 to 2018. In Jilin Province, Baicheng City and Changchun City have the highest similarity (*C* = 0.990), while Baishan City and Songyuan City have the lowest *C*-values (*C* = 0.511). Except for the above cases, the similarity coefficients among the cities in Jilin Province were higher than 0.8. The similarity coefficient between cities in Jilin Province and Chuxiong Yi Autonomous Prefecture was moderate (*C* = 0.506–0.901), and the similarity with Xiamen City was a little higher (*C* = 0.800–0.905). The similarity of occupational composition of HEV cases between the remaining 11 cities in Jiangsu Province was largely above 0.9, except for Nantong City and Suzhou City. Suzhou and Nantong Cities had the lowest similarity with most cities in Jiangsu Province (*C* < 0.4). The cities of Nanjing City and Wuxi City in Jiangsu Province had higher similarity coefficients with cities of Jilin Province (*C* = 0.8497–0.960), and Xiamen City of Fujian Province (*C* = 0.892–0.924). The occupational distribution among the 11 cities in Jiangsu Province was similar to that of Chuxiong Yi Autonomous Prefecture (*C* > 0.9). The similarity index values between Xiamen City and Chuxiong Yi Autonomous Prefecture differed significantly (*C* = 0.455).

**Table 3:** Matrix of occupational similarity in the number of cases of hepatitis E in 24 cities (2008–2018).

**Year**	**Baicheng**	**Baishan**	**Jilin**	**Liaoyuan**	**Siping**	**Songyuan**	**Tonghua**	**Yanbian**	**Changchun**	**Nanjing**	**Wuxi**	**Xuzhou**	**Changzhou**	**Suzhou**	**Nantong**	**Lianyungang**	**Huaian**	**Yancheng**	**Yangzhou**	**Zhenjiang**	**Taizhou**	**Suqian**	**Xiamen**	**Chuxiong**
Baicheng City	1.000																							
Baishan City	0.852	1.000																						
Jilin City	0.975	0.775	1.000																					
Liaoyuan City	0.929	0.914	0.891	1.000																				
Siping City	0.888	0.636	0.891	0.762	1.000																			
Songyuan City	0.834	0.511	0.846	0.658	0.658	1.000																		
Tonghua City	0.953	0.747	0.947	0.865	0.865	0.926	1.000																	
Yanbian Korean Autonomous Prefecture	0.846	0.916	0.748	0.948	0.948	0.543	0.543	1.000																
Changchun City	0.990	0.834	0.969	0.910	0.910	0.825	0.825	0.834	1.000															
Nanjing City	0.903	0.886	0.877	0.827	0.675	0.627	0.781	0.768	0.921	1.000														
Wuxi City	0.940	0.810	0.961	0.901	0.821	0.781	0.929	0.768	0.947	0.885	1.000													
Xuzhou City	0.731	0.377	0.793	0.562	0.900	0.942	0.823	0.401	0.720	0.529	0.707	1.000												
Changzhou City	0.925	0.647	0.957	0.820	0.951	0.939	0.970	0.670	0.915	0.744	0.918	0.896	1.000											
Suzhou City	0.539	0.214	0.596	0.385	0.766	0.825	0.651	0.253	0.525	0.336	0.510	0.938	0.729	1.000										
Nantong City	0.539	0.214	0.596	0.384	0.765	0.825	0.651	0.252	0.525	0.335	0.509	0.937	0.729	1.000	1.000									
Lianyungang City	0.827	0.484	0.875	0.665	0.949	0.974	0.905	0.508	0.815	0.625	0.799	0.982	0.957	0.868	0.868	1.000								
Huaian City	0.692	0.338	0.749	0.523	0.886	0.935	0.798	0.374	0.680	0.476	0.662	0.996	0.868	0.959	0.959	0.968	1.000							
Yancheng City	0.708	0.359	0.763	0.538	0.903	0.947	0.818	0.385	0.693	0.493	0.682	0.996	0.881	0.949	0.949	0.975	0.998	1.000						
Yangzhou City	0.765	0.408	0.823	0.593	0.918	0.964	0.860	0.434	0.758	0.564	0.750	0.995	0.924	0.908	0.907	0.993	0.987	0.991	1.000					
Zhenjiang City	0.883	0.601	0.931	0.727	0.944	0.949	0.946	0.556	0.878	0.747	0.897	0.915	0.970	0.753	0.753	0.963	0.886	0.903	0.943	1.000				
Taizhou City	0.772	0.423	0.830	0.606	0.925	0.966	0.874	0.443	0.763	0.570	0.767	0.990	0.932	0.897	0.897	0.993	0.981	0.988	0.998	0.952	1.000			
Suqian City	0.667	0.319	0.721	0.498	0.877	0.928	0.781	0.353	0.653	0.449	0.637	0.989	0.848	0.969	0.969	0.956	0.998	0.997	0.979	0.870	0.973	1.000		
Xiamen City	0.809	0.838	0.701	0.764	0.633	0.620	0.757	0.822	0.822	0.829	0.763	0.432	0.651	0.288	0.288	0.533	0.409	0.428	0.480	0.622	0.493	0.396	1.000	
Chuxiong Yi Autonomous Prefecture	0.730	0.370	0.794	0.548	0.893	0.939	0.814	0.386	0.722	0.539	0.699	0.998	0.889	0.931	0.931	0.980	0.992	0.991	0.992	0.914	0.985	0.984	0.427	1.000

### The Results of Cluster Analysis on Occupational Types in the 24 Cities

**Figure 5:**
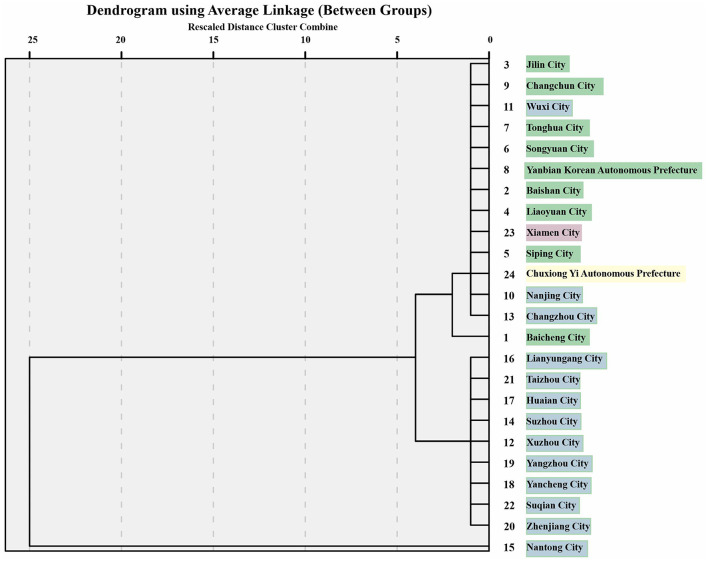
Diagram of cluster analysis results of occupational incidences in 24 cities (2008-2018).

From the clustering result chart of Figure 5, when the cities were divided into two categories, Nantong City was in its own group, while the other 23 cities were in another group.

When the cities were divided into three categories, Nantong City was still divided into a separate group, nine cities in Jilin Province, Wuxi City, Nanjing City, Changzhou City, Xiamen City, and Chuxiong Yi Autonomous Prefecture were grouped together, and the remaining nine cities, including Yancheng City and Xuzhou City in Jiangsu Province, were grouped together.

When the cities were divided into four categories, the results were consistent with those of when they were divided into three categories; the only difference was that Baicheng City was classified as a separate group.

### The Comparisons of Results Between New Method and Cluster Analysis

Most of the cities in Jilin province were close in similarity and diversity and were classified into the same group. The similarity coefficients between Nanjing and Baishan, Jilin, Liaoyuan, and Changchun (*C* > 0.8), and Nanjing and Xiamen (*C* = 0. 924) are similar, which is consistent with the results of the cluster analysis.

In the cluster analysis, Baishan City and Songyuan City, which do not have high diversity and similarity, were nevertheless placed in the same category, and it was the same for Xiamen City and Chuxiong Yi Autonomous Prefecture. The similarity index between Xiamen, a coastal city, and Chuxiong Yi Autonomous Prefecture, an inland region, was not high (*C* = 0.455), yet they were grouped together in the clustering. Nantong city and Suzhou city had the highest similarity (*C* = 1.000); however, the cluster analysis did not provide enough information about why these two cities were not grouped into the same category. Similarly, 11 cities in Jiangsu Province have similar occupational distribution (*C* > 0.9) to Chuxiong Yi Autonomous Prefecture, yet Chuxiong Yi Autonomous Prefecture is grouped with three other cities in Jiangsu Province, namely Nanjing, Wuxi, and Changzhou, by cluster analysis.

## Discussion

### Epidemiological Characteristics Analysis

The incidence of HEV infection varied among the 24 cities in the four regions. One study (12) also confirmed that the incidence of HEV infection was lower in the central (Jilin Province) and western (Chuxiong Yi Autonomous Prefecture) regions than in the eastern region (Jiangsu Province, Xiamen City) in China from 2004 to 2017. There are more river systems and frequent floods in the southern region, which may contribute to transmission via dirty water. In addition, it may also be related to the improvement of surveillance levels, the popularization of diagnostic reagents (26) and HEV mutation (27) in southern China, where economic and demographic structures are more complex. The high incidence in middle-aged and elderly people may be associated with the natural history of the disease. This is consistent with the finding that population antibody levels increased with age (28, 29). According to the WHO report, the infection rate in children is low, and the affected population is mainly adults (30). The majority of HEV patients were male, as expected, since males had fewer chores than women; thus, they had less exposure to dirty water and animals.

We found that farmers accounted for a large proportion of patients. This is consistent with the results of several epidemiological studies (12, 31). Farmers are easily exposed to contaminated water and are in close contact with animals for living in rural areas. HEV contamination of pig manure and water sources can be accompanied by potential transmission of contaminated agricultural or seafood to humans through the food chain. Recently, a systematic review identified that living in rural areas is a risk factors for anti-HEV IgG positivity (32). Housework and unemployed people are also exposed to animal viscera, and sewage during cooking, so a significant number of groups could be infected. Retirees and elderly individuals are easily infected because of their poor status and immunity (33).

Several occupational incidences are at an intermediate level for balancing occupational exposure and hygiene prevention Compared with farmers, migrant workers live in cities and have less direct contact with animals. Medical staff, teachers, and students have a relatively small incidence of infections due to the implementation of disinfection measures at hospitals and schools. The routes of environment-to-human and animal-to-human transmissions are difficult to achieve.

We found that there were few or no records of several occupational types. For people in the catering industry, caregivers, and babysitters, they undergo health examinations by the local Centre for Disease Control and Prevention before entering their work, which prevents the spread of HEV to some extent. We did not observe any diaspora children or childcare group since they are not as susceptible as adults and have access to the meticulous care; herder is rare occupation, and there may exist many unreported cases. The fishing (boat) group is not susceptible to HEV, indicating that being exposed to seafood is not as contagious as other animals.

### Analysis of HEV Occupational Incidence in the 24 Cities by the New Method

The occupational distribution of HEV cases in Jiangsu Province was more balanced than in the other three regions. Unlike most cities, Baicheng and Nanjing cities have developed economies and large populations, so the dominant occupational type is not farmers. The relatively even distribution of HEV-affected occupations in Liaoyuan, Nanjing, and Xiamen cities may be due to demographic reasons, and the cities are richer in occupational types.

We believe that the economic environment is a key factor in determining occupational similarity. Nanjing and Wuxi, two cities located in the southern part of Jiangsu province, have a developed economy and fewer people are engaged in HEV-related high-risk occupations, such as agriculture, compared to other cities. Similarly, Xiamen has a well-developed economy and a high degree of similarity with the Nanjing and Wuxi cities. The less economically developed Chuxiong Yi Autonomous Prefecture had lower occupational similarity with Nanjing, Wuxi, and Xiamen cities. The high incidence in the 24 cities was concentrated in the central urban areas, where there are more employment opportunities, more frequent human contact, and therefore a greater potential for transmission.

As far as the differences in *N*-values within Jiangsu province, such as with only two occupations in the HEV-affected population in Suzhou and Nantong, we speculate that this may be related to inaccurate and under-reported disease reporting, as well as the uneven degree of development within the same province. The results showed that the occupational distribution among HEV cases was more diverse in Liaoyuan, Nanjing, and Xiamen cities. In contrast, the opposite was true for the Nantong and Chuxiong Yi autonomous prefectures.

### The Cluster Analysis of HEV Occupational Incidence in the 24 Cities

When each city is divided into two categories, it shows the difference in the distribution of occupational morbidity. Nantong City is classified as a separate category, because the incidence of hepatitis E in Nantong is entirely contributed by the peasant population. When divided into three categories, we can see the differences between cities within the same province. For example, three cities in Jiangsu Province were classified in one category with all cities in Jilin province, Xiamen city, and Chuxiong Yi Autonomous Prefecture, while the remaining 10 cities in Jiangsu Province were in another category. These cities all had similar levels of hepatitis E prevalence and similar occupational compositions of high and low prevalence.

When the cities were divided into four categories, we can also find small differences in cities within the same province. For example, the city of Baicheng in Jilin Province is separated from the other eight cities in the same province, probably because Baicheng has only 10 occupations for the HEV-affected population, while all other cities have 12 or more types.

From the results of the cluster analysis, we were able to classify the cities according to the incidence of the type of occupation, as well as with increasing grouping, we were able to find differences between cities within the same province.

### Comparisons Between the New Method and Clustering Analysis

When analyzing the occupational composition of hepatitis E across regions and thus classifying cities, both methods based on the cluster analysis method and the new method yielded similar results in most cases and a few opposite results. We believe there may be several reasons for this. First, the principles of the two methods are different. The clustering method we used was analyzed in the form of defining the distance between classes, and the results obtained may be concise. The new method is a comprehensive analysis of diversity, balance, similarity, and other levels with the help of six major indicators to obtain rich results. Second, the new method does not establish a good connection between the values of each indicator and the specific criteria. For example, for the similarity coefficient *C*-value, we think that when the *C*-value is higher than 0.8, then the two cities are more similar; if the *C*-value is lower than 0.5, then the similarity is lower. Further research could focus on the criteria system to improve classification accuracy.

We confirmed the validity in cross-regional disease occupational composition analysis, which is an extension of the method from microbial level classification to population level. The method has good feasibility and applicability, and more detailed outcome indicators can be obtained.

### Suggestions for Prevention and Control Measures

First, we need to strengthen health education on hepatitis E prevention and control for various occupational risk groups. Awareness of hepatitis E is significantly lower than that of hepatitis B and C, especially for key occupational groups, such as farmers engaged in livestock (pig) and poultry-related farming or slaughtering, as well as retirees, housework and unemployment groups.

Second, it is necessary to control the transmission of viral hepatitis by frequently testing HEV in workers of related occupations. For example, the rate of positive IgM antibodies to HEV can be used as a signal indicator.

Third, the main strategies to deal with HEV in China at this stage are the development of HEV vaccines and the improvement of laboratory diagnosis rates. One study considered the strategy of HEV vaccination in women of childbearing age (34), and public health professionals recommended promoting HEV vaccine in Shanghai (35). We believe there is a need to consider the strategy of HEV vaccination in high-risk occupational groups, such as farmers, to effectively reduce the disease burden.

## Limitations

First, the regions we chose were not random, which may lead to deviations. This was a preliminary study. In the future, if possible, we will use the disease data from more regions for in-depth studies.

Second, because not all cases were genotyped in the laboratory, we could not include genotypes for a detailed study due to the availability of data.

Third, we could not analyze the disease severity of hepatitis E. Cases of hepatitis E are largely common and mild, with few critical illnesses and deaths. The latter tends to be common in pregnant women and in the older age group. If pregnant women are infected, serious consequences are associated not only with high mortality in the late fetal period, but also with the occurrence of preterm birth and a high probability of vertical transmission to offspring (36, 37). However, the incidence in these populations is low.

## Conclusions

In China, the factors influencing the prevalence of hepatitis E are living in the south, working as farmers, being middle-aged or elderly, and being male. The 24 cities we studied were highly diverse and moderately similar in terms of the occupational distribution of patients with hepatitis E. We confirmed the validity of the new method in classifying cities according to their occupational composition by comparing it to the clustering analysis.

## Data Availability Statement

The raw data supporting the conclusions of this article will be made available by the authors, without undue reservation.

## Author Contributions

TC, XZ, and QZ designed the study. SY, JR, XC, ZZ, CL, SL, YZ, YW, JX, MY, and XL collected the data. SY, JR, XC, ZZ, MW, and ZL analyzed the data. SY and JR wrote the manuscript. All authors have read and approved the final manuscript.

## Funding

This study was partly supported by the Bill & Melinda Gates Foundation (INV-005834), the Science and Technology Program of Fujian Province (No. 2020Y0002), the Xiamen New Coronavirus Prevention and Control Emergency Tackling Special Topic Program (No. 3502Z2020YJ03), and the XMU Training Program of Innovation and Entrepreneurship for Undergraduates (2019Y1497, 2019Y1500, and 2019Y1501). The funders had no role in the study design, data collection and analysis, decision to publish, or preparation of the manuscript.

## Conflict of Interest

The authors declare that the research was conducted in the absence of any commercial or financial relationships that could be construed as a potential conflict of interest.

## Publisher's Note

All claims expressed in this article are solely those of the authors and do not necessarily represent those of their affiliated organizations, or those of the publisher, the editors and the reviewers. Any product that may be evaluated in this article, or claim that may be made by its manufacturer, is not guaranteed or endorsed by the publisher.
